# Gypsy, Roma and Traveller populations and mental health in the UK: a need for real working together and co-production of services

**DOI:** 10.1192/bji.2024.14

**Published:** 2024-08

**Authors:** Radha Kothari, Amy Ward, Derek Tracy

**Affiliations:** 1 Principal Clinical Psychologist and Clinical Lead, Central and North West London Health and Justice Service, His Majesty's Prison/Young Offender Institution Feltham, London, UK; 2 National Traveller Accommodation Policy Officer, Irish Traveller Movement, Dublin, Ireland; 3 Chief Medical Officer, West London NHS Trust, London, UK

**Keywords:** Traveller, gypsy, stigma and discrimination, mental health services, transcultural psychiatry

## Abstract

Gypsy, Roma and Traveller (GRT) communities have considerably worse mental health outcomes than the general population and many other ethnic minority groups. We argue that there is a dynamic, interplaying ‘accessibility mismatch’, resulting in a failure of healthcare services to adequately understand and work with GRT communities in a meaningful way. The consequences are limited engagement and poor health outcomes. Contact with services is often at crisis points, such as in forensic services, which reinforces existing prejudice. Research is limited, and therefore so is the evidence base. It is critical that the UK's National Health Service takes a culturally informed approach to co-produce services that are accessible and responsive to GRT communities. Here we offer practical actions that healthcare organisations can undertake to help redress imbalances and increase equity of healthcare outcomes for these overlooked populations.

Gypsy, Roma and Traveller, or ‘GRT’, populations is a recognised umbrella term often used generically to describe communities with a historical and cultural nomadic tradition. This includes heterogeneous groups of English and Welsh Gypsies, Romany, Irish and Scottish Travellers, Roma, Romani, French Manush, Sinti and others. We adopt the term GRT in this article, but emphasise its limitations, including that it risks counterproductively clustering together culturally rich, diverse and heterogeneous communities, whose needs may be quite different, and thus GRT paradoxically may reinforce stigma for some.

Nevertheless, taking these caveats into account, evidence indicates a shocking disparity in healthcare outcomes, with GRT communities experiencing considerably higher rates of mental health difficulties and dramatically lower life expectancy compared with the general populationr in Ireland and the UK.^1^ In the UK, recent efforts made to co-produce integrated healthcare services with patients, which emphasise prevention and reduction of population health inequalities, appear not to be extended to GRT communities. This is evidenced by the fact that initial contact with healthcare is often in substance misuse services, crisis teams (often at times of significant self-harm) and in prison healthcare services.^2^ The standard approach to engagement and service provision is clearly inadequate and failing. This is exacerbated by significant prejudices among healthcare professionals that mirror those of the wider population.^3,4^ In comparison to the collaborative working that is increasing with other ethnically marginalised population, there is a lack of proactive attempts by healthcare professionals and services to effectively understand and work collaboratively with GRT communities. In fact, we contest that (with some exceptions), the National Health Service (NHS) has done little to suggest that it is an organisation that appreciates or is attempting to redress the critical levels of health inequalities that exist for GRT populations.

## Why is this happening?

As with many discriminated against and marginalised populations, stereotyped assumptions and prejudice among professionals have led to GRT communities feeling shamed by, and having a mistrust of, healthcare professionals. Unsurprisingly, this prevents engagement, which in turn perpetuates a lack of understanding among professionals, resulting in continued culturally uninformed practice. Additionally, as is the case with many cultures that are collectivist rather than individualistic in nature, people from GRT communities often face a high degree of both internalised and externalised cultural stigma associated with experience of mental health difficulties. This can hinder proactive engagement with services when difficulties first emerge,^5^ making it more likely that people will first present to services at a time of crisis. Expectations such as a permanent residential address, minimum literacy requirements or access to and familiarity with information technology are additional barriers to access that appear to affect GRT communities more severely. The culturally informed flexibility that services would require to support access to healthcare is historically lacking, which contributes to the isolation of GRT communities from broader society.^2^ In summary, people from GRT backgrounds are likely to be deterred by internal and external threats that come with help-seeking, such as judgement from one's own community and prejudice from broader society, in addition to having to consider practical barriers that could be considered institutionally racist.

The aim of this paper is to explore how the NHS might take practical steps to redress this inequality: we re-emphasise that needs of different GRT groups, and of individuals, may vary widely.

## What it looks like in practice: a crisis interface

For the reasons detailed above, people from GRT or culturally nomadic communities often seek help for the first time when at points of crisis.^4^ They are therefore more likely to make contact with healthcare professionals in settings such as emergency departments for mental or physical health crises or substance misuse emergencies, in prisons and forensic settings following forced detainment, or through crisis teams when they experience suicidal ideation or self-harm.^6^ Support at crisis points is necessary and can be viewed as an opportunity to engage individuals from GRT communities who have not previously sought help; however, it is generally accepted that prevention or early intervention is more effective for physical and mental health difficulties and substance misuse.^2^ Contact with healthcare professionals at crisis points or in forensic environments can also emphasise existing power dynamics between ‘clinician’ and ‘patient’, and this is likely to be more keenly felt by those from GRT communities owing to the prejudice, existing barriers and stigma described above. Overall, the experience of engaging with healthcare only at times of crisis lends itself to reinforcement of biases on both sides of the interaction.

The impact of adverse childhood experiences and intergenerational trauma among GRT communities is under-researched, poorly understood and therefore given little consideration, particularly in ‘crisis’ type settings. The long-term harms that can come with this kind of developmental trauma are now well-known and it is possible that this is a mechanism through which the particularly poor outcomes observed within GRT communities are perpetuated from one generation to the next. Over recent years, there has been an increase in trauma-informed interventions designed to prevent, or at least limit, intergenerational transmission of poor physical and mental health outcomes (e.g. Public Health Network Cymru's educational video at https://www.youtube.com/watch?v=XHgLYI9KZ-A). As with other interventions, these are less likely to reach GRT communities, and there has been woefully little work done to co-produce psychoeducation, prevention or early intervention approaches that would be more accessible and effective.

## What can we do?

### Naming the issue and starting co-production

Change needs to occur, and we believe that this can and must happen in several different ways. To begin with, the issue needs to be named and recognised: this is a national, organisational and individual challenge.^2^ Healthcare professionals and services need to start from a position of humility and outreach, and appreciate historical and current societal prejudices and injustices. In the UK, the prioritisation of local population health inequalities offers, at least in principle, a potential driver for change.^7^ It will be critical to raise interest among healthcare professionals, but there must be true co-production with GRT communities to identify priorities and methods of working. This is vital to prevent assumptions, bias and prejudice having a negative impact and future service development being ineffective and inaccessible. We were pleased to see, during the publication process of this article, that the NHS Race and Health Observatory published a national guide on identifying best practice.^6^ Endeavours such as this are essential to raise the national profile of the issue.

### Moving from crises to culturally informed preventive approaches

There is a need to grow our culturally informed practice across the NHS, including recognition and embracing of the heterogeneity and variation within GRT communities. Considered optimistically, engagement at times of crisis could offer an opportunity for healthcare professionals to meaningfully explore biases and power dynamics, which might contribute to improved understanding on both sides and increased trust. This should also improve relationships with professionals among GRT communities. As well as providing appropriate provision at crisis points, organisations and services must work with GRT communities to co-produce services that proactively tackle health inequalities through public health outreach, psychoeducation and increased engagement from onset of difficulties. It is important to address societal inequalities, often experienced from birth, and to address the impact of GRT-specific adverse childhood experiences and intergenerational trauma.

There are particular opportunities working with children and young people and their parents, where historically experiences have often been aversive, including perceived hostile interventions from social services, forced removal of children and so forth. Working collaboratively with GRT communities should develop a more informed, supportive and compassionate approach. In turn, this should foster less pejorative judgement and lean less into existing power dynamics. Ultimately, the aspiration is that this will increase opportunities for improved relationships with professionals from an early age, which could extend throughout the lifetime. In considering intergenerational trauma, rates of mental health difficulties in children and the potential for preventive work, it seems an opportune scenario to build positive relationships. This early building of such relationships would afford opportunities to provide effective health interventions throughout the lifetime, from pregnancy, throughout childhood and into young, middle and older adulthood.

### Working on professional and organisational biases

To influence real change, organisations and leaders must address the power imbalance that is too often created and maintained by prejudice and preconceptions. Healthcare professionals are also members of the public and often of local populations. They are liable to share associated biases, from the unconscious to frank discriminatory and prejudicial behaviour. It is our own belief that for some, it remains perhaps more societally acceptable to have such attitudes about GRT communities than would be ‘sanctioned’ for other marginalised populations. We lack good data on the number of health professionals from, or representative of, GRT communities. In our anecdotal experience, it is far below that of other marginalised groups in the UK and Ireland. Recent data from NHS England's Workforce Race Equality Standard (WRES) report show far greater rates of bullying and harassment and greater discrimination suffered by staff from GRT communities than any other marginalised groups.^9^ Data from Ireland suggest that perhaps only 1% of Irish Travellers complete a third level education,^10^ and systemic inequalities in education may also be a hindering factor.

There is an absence of people from GRT backgrounds in key positions in statutory agencies, trade unions, health boards and places of political representation. However, we note that figures from the Traveller Movement indicate that 76% of Travellers in the UK have hidden their ethnicity in order to avoid discrimination or prejudice.^11^ Contemporary services speak of valuing diversity, but the limited data we have show a failure to integrate GRT communities into our workforce and into our services. It is not clear why this should be any more difficult or culturally complex than for any other marginalised population. In our opinion, it speaks to a profundity of prejudice and an inability or unwillingness to recognise this problem. In current NHS practice, this is – or should be – seen as an organisational and societal failure of engagement rather than a ‘failure’ of a community. We need to better understand the factors that limit understanding or engagement of healthcare professionals, and there would appear to be fertile ground for joint learning in this regard.

### Building trusting relationships

Relationships are key and attempts to seek help should be treated as opportunities to engage and improve ‘relationships with help’ and trust in professionals. There are opportunities for all of us providing care to link with patients and communities to ensure that approaches are not limited by standard care protocols. This might include removing barriers to making referrals because of lack of an established address and so forth. Relationships and trust allow communities to feel heard and understood. Advocates and ambassadors should be involved in promoting best practice and engaging communities, potentially acting as a bridge for understanding and increasing trust. There is potential for NHS England's integrated care systems (ICSs) to commission specialist services in areas with larger GRT populations and substance misuse services. Although ICSs do not currently apply to prisons, attention should be given to providing culturally informed physical and mental health assessments and interventions in this setting. There are examples of additional resources and culturally appropriate help to pull on: for example, culturally adapted talking therapies in South Asian populations^8^ and related work with diabetes care.

### Having GRT-led conversations

We believe that there is a critical role for GRT-led conversations, both between communities and services to improve understanding and relationships and also within GRT communities to explore the impact of isolation and stigma on mental and physical health outcomes. In our anecdotal experience, despite the stark statistics speaking to the trauma of GRT communities, individuals from those backgrounds often say that they do not feel they would be believed even if they did speak about their trauma experiences; that if they are believed then their community as a whole would be judged; or that those external to the community would not fully understand. We believe that there is a need for training and upskilling for healthcare professionals, to be aware of their own bias and prejudice and also to be more culturally informed. There are also opportunities for community ambassadors, trained and supported by culturally informed physical and mental health organisations, to help increase knowledge and destigmatise mental illness within GRT communities. There are rich contemporary examples to draw on, such as recent work in training barbers of Black ethnicity in the UK on mental health issues.^12^

## Conclusion

There has been a lack of broader discussion on the nuances of GRT mental health. No one size fits all for these diverse and rich communities, but in comparison with many other marginalised groups, care remains substandard, engagement poor, and prejudice and bias appear to be hidden in plain sight. Indeed, we would argue that prejudicial views from services and professionals are common, unchallenged and not even identified as problematic. Those of GRT background are often not perceived as having culturally specific needs – or needs that vary among them – and are somehow separated from other immigrant or ethnically marginalised communities. Perhaps an appropriate analogy is with Australian and American First Nation populations, who continue to face enormous racism and exclusion^13^ that has historically been state sanctioned.

The needle has not moved in recent times. There are dangers that figures on poor outcomes are just reiterated, reinforcing a population ‘otherness’ and a deficit model of people seen only through a lens of crisis interfaces. We lack good data, although there is an emerging literature, including both a recent review of the topic^1^ and policy documents by both Traveller and Gypsy^2^ and NHS groups.^6^ We need to move beyond simple reporting and must work collaboratively with communities, accounting for the diverse sociocultural and ethnic backgrounds under the umbrella term of ‘GRT’. It is clear to us that services, not communities, need to stretch, but there is also rich space to grow conversations with GRT communities.

There is much that we do not know, and as yet we have not even formulated the key research, care or policy objectives. Perhaps a good starting point would be to ask ourselves why this is the case.

## Data Availability

Data availability is not applicable to this article as no new data were created or analysed in this study.
